# Social jetlag, a novel predictor for high cardiovascular risk in blue‐collar workers following permanent atypical work schedules

**DOI:** 10.1111/jsr.13380

**Published:** 2021-05-04

**Authors:** Sara Gamboa Madeira, Cátia Reis, Teresa Paiva, Carlos Santos Moreira, Paulo Nogueira, Till Roenneberg

**Affiliations:** ^1^ Instituto de Saúde Ambiental (ISAMB) Faculdade de Medicina Universidade de Lisboa Lisbon Portugal; ^2^ Family Health Unit Mactamã Administração Regional de Saúde de Lisboa e Vale do Tejo (ARSLVT) Lisbon Portugal; ^3^ Católica Research Centre for Psychological Family and Social Wellbeing (CRC‐W) Universidade Católica Portuguesa Lisbon Portugal; ^4^ Faculdade de Medicina Instituto de Medicina Molecular João Lobo Antunes Universidade de Lisboa Lisbon Portugal; ^5^ Sleep Medicine Center (CENC) Lisbon Portugal; ^6^ Comprehensive Health Research Center (CHRC) Nova Medical School Faculdade de Ciências Médicas Lisbon Portugal; ^7^ Medicine Clinic I Faculdade de Medicina Universidade de Lisboa Lisbon Portugal; ^8^ Área Disciplinar Autónoma de Bioestatística Faculdade de Medicina Universidade de Lisboa Lisbon Portugal; ^9^ Instituto de Medicina Preventiva e Saúde Pública Faculdade de Medicina Universidade de Lisboa Lisbon Portugal; ^10^ Institute and Polyclinic for Occupational‑ Social‑ and Environmental Medicine LMU Munich Munich Germany; ^11^ Chronsulting UG Dietersburg Germany

**Keywords:** circadian misalignment, MCTQ, SCORE, shift‐work

## Abstract

Cardiovascular diseases cause >4 million deaths each year in Europe alone. Preventive approaches that do not only consider individual risk factors but their interaction, such as the Systematic COronary Risk Evaluation (SCORE), are recommended by European guidelines. Increased cardiovascular risk is associated with shift‐work, surely interacting with the concurrent conditions: disruption of sleep, unhealthy behaviours, and circadian misalignment. Social jetlag (SJL) has been proposed as a way to quantify circadian misalignment. We therefore investigated the association between SJL and cardiovascular health in a cross‐sectional observational study involving blue‐collar workers, who either worked permanent morning, evening, or night shifts. Sociodemographic, health and productivity data were collected through questionnaires. Blood pressure and cholesterol were measured and the cardiovascular risk was estimated according to the relative risk SCORE chart. Bivariate analysis was performed according to the cardiovascular risk and the relationship between SJL and high cardiovascular risk was analysed through logistic regression. Cumulative models were performed, adjusted for various confounding factors. After 49 exclusions, the final sample comprised 301 workers (56% males; aged <40 years, 73%). Mean standard deviation (*SD*) SJL was 1:57 (1:38) hr (59.4% ≤2 hr). Cardiovascular risk was high in 20% of the sample. Multivariate analysis revealed SJL to be an independent risk factor for high cardiovascular risk. Each additional hour of SJL increased this risk by >30% (odds ratio 1.31, 95% confidence interval 1.02–1.68). This is the first study indicating that SJL potentially increases cardiovascular risk, and suggests that sleep and individual circadian qualities are critical in preventing negative health impacts of shift‐work.

## 1 INTRODUCTION

Ischaemic heart disease and stroke are expected to be the two leading causes of premature death by 2040, despite cost‐effective medications being available for major risk factors (IHME, 2018). As cardiovascular diseases (CVDs) share similar pathophysiological mechanisms, approaches that do not only consider individual risk factors but their interaction, such as the Systematic COronary Risk Evaluation (SCORE), are recommended by the European guidelines on CVD prevention (Piepoli et al., 2016). The SCORE is based on large, representative European cohorts (Conroy et al., 2003); the SCORE chart estimates the 10‐year‐risk of dying from a CVD based on five factors: age, gender, smoking, systolic blood pressure (SBP), and total cholesterol (TC). Because age has the strongest impact on CVD risk, young people (i.e. aged <40 years) pose a particular challenge because their low absolute risk may conceal a high relative risk. Therefore, a relative risk SCORE chart has been established, considering only the modifiable risk factors: smoking, SBP, and TC (Piepoli et al., 2016). While risk is a continuum, and there are no universally applicable thresholds, the intensity of interventions should increase with increasing risk estimation (Mach et al., 2020).

Our “24/7” societies increasingly involve atypical work schedules, which affect >20% of the European working population (Eurofound, 2017). A systematic review, including >2 million people, concluded that shift‐work is associated with a 23% higher risk of ischaemic heart disease and 5% more for stroke (Vyas et al., 2012). Different pathways have been proposed to explain the link between shift‐work and health, namely disruption of sleep, unhealthy behaviours, and circadian misalignment (Kecklund & Axelsson, 2016). Circadian misalignment can be defined as the difference between the “social clock” (e.g. work schedules) and the “biological clock”. Entrainment of biological clocks to the environmental light–dark cycle can be very different among individuals (i.e. different chronotypes) (Roenneberg & Merrow, 2016). Chronotype, as an expression of how individual biological clocks entrain with the 24‐hr cycle, is the key concept behind the Munich ChronoType Questionnaire (MCTQ) (Wirz‐Justice et al., 2003). The MCTQ uses sleep‐timing, specifically its mid‐point on work‐free days to estimate chronotype and compares it to the mid‐sleep point on workdays to calculate social jetlag (SJL). SJL is a way to quantify circadian misalignment (Wittmann et al., 2006). Regarding CVD risk factors, a systematic review found a positive association between SJL and smoking, heart rate (HR), exercise, body mass index (BMI), and triglycerides, but not with cholesterol and blood pressure (BP) (Castilhos Beauvalet et al., 2017).

Previous studies showed that, among workers following rotating shift schedules, earlier chronotypes working night shifts had shortened sleep, higher levels of SJL and sleep disturbances. A similar pattern was noted for later chronotypes during morning shifts (Juda et al., 2013). Aligning work shifts with individual chronotype, minimising SJL, has already demonstrated to improve wellbeing and sleep (Vetter et al., 2015). In a three‐shift rotating schedule, the individual disadvantages occur only one‐third of the work hours, individualised shift assignments are therefore even more relevant when atypical schedules are permanent. To our knowledge, studies assessing the role of SJL among shift‐workers under permanent schedules are lacking. In the present study, we therefore aimed to investigate the association between SJL and cardiovascular health in a population of blue‐collar workers following permanent atypical schedules.

## 2 METHODS

### 2.1 Participants and ethics

The study population was comprised of working adults performing manual picking activity in a retail facility, near Lisbon, Portugal. All workers were invited to participate (census approach) and gave their written informed consent as volunteers. No rewards were given, or privileges withdrawn for workers participating in the study. The company did not participate in the study design and the study protocol was approved by the Ethics Committee of the Medical School at the University of Lisbon. Workers performed permanent (non‐rotational) schedules comprising morning (06:00–15:00 hours), evening (15:00–24:00 hours) or night shift (21:00–06:00 hours). All participants accepted to participate and a total of 350 workers took part in the study. In the present analysis, exclusion criteria were part‐time contract, <3 months within the present schedule, having an extra job or performing standard schedule (*n* = 36). As our sample comprised very different schedules, we excluded the extreme outliers (>×3 the interquartile range [IQR]) above the third quartile for SJL (*n* = 11). Two individuals refused the collection of blood for cholesterol analysis; therefore, the final sample was composed of 301 workers (Tables 1 and 2).

**TABLE 1:** Sociodemographic and clinical features of all participants and according to the cardiovascular disease (CVD) relative risk, for categorical variables

Variable	All participants	Relative risk CVD		*p*
		High risk	Others	
	*N* (%)	*n* (%)	*n* (%)	
Participants	301 (100)	61 (20.3)	240 (79.7)	
Sex				
Male	169 (56.1)	27 (44.3)	142 (59.2)	.051
Female	132 (43.9)	34 (55.7)	98 (40.8)	
Age groups, years				
≤25	79 (26.2)	7 (11.5)	72 (30.0)	**<.001**
25–40	141 (46.8)	25 (40.9)	116 (48.3)	
≥40	81 (26.9)	29 (47.5)	52 (21.7)	
Education groups, years				
≤12	264 (87.7)	56 (91.8)	208 (86.7)	.383
>12	37 (12.3)	5 (8.2)	32 (13.3)	
Schedule				
Morning	153 (50.8)	32 (52.5)	121 (50.4)	.179
Evening	120 (39.9)	20 (32.8)	100 (41.7)	
Night	28 (9.3)	9 (14.8)	19 (7.9)	
SDur workdays, hr				
>6	181 (60.1)	27 (44.3)	154 (64.2)	.**007**
≤6	120 (39.9)	34 (55.7)	86 (35.8)	
Sleep quality				
≥Good	205 (68.1)	39 (63.9)	166 (69.2)	.529
≤Poor	96 (31.9)	22 (36.1)	74 (30.8)	
Social jet lag				
≤2	179 (59.4)	31 (50.8)	148 (61.7)	.065
2−4	98 (32.6)	21 (34.4)	77 (32.1)	
≥4	24 (8.0)	9 (14.8)	15 (6.2)	
Physical exercise, min/week				
≥150	56 (20.4)	3 (9.8)	53 (31.7)	.**002**
<150	219 (79.6)	55 (90.2)	164 (68.3)	
Alcohol, drink/day				
≤1	272 (90.4)	54 (88.5)	218 (90.8)	.762
>1	29 (9.6)	7 (11.5)	22 (9.2)	
Caffeine, drinks/day				
≤3	212 (70.4)	30 (49.1)	182 (75.8)	**<.001**
>3	89 (29.6)	31 (50.8	58 (24.2)	
BMI, kg/m^2^				
<25	187 (62.1)	39 (63.9)	148 (61.7)	.859
≥25	114 (37.9)	22 (36.1)	92 (38.3)	
Smoking				
No	149 (49.5)	1 (1.6)	148 (61.7)	**<.001**
Yes	152 (50.5)	60 (98.4)	92 (38.3)	
Hypertension, blood pressure, mmHg				
<140 and 90	269 (89.4)	221 (92.1)	48 (78.7)	.**005**
≥140 or 90	32 (10.6)	19 (7.9)	13 (21.3)	
Hypercholesteraemia, total cholesterol, mg/dl				
<190	154 (51.2)	8 (13.1)	146 (60.8)	**<.001**
≥190	147 (48.8)	53 (86.9)	94 (39.2)	

Abbreviations: BMI, body mass index; CVD, cardiovascular disease; SDur, sleep duration.

Relative frequencies in CVD risk categories refer to the prevalence within “high‐risk CVD” and “others” groups. Data is presented as absolute (*n*) and relative (%) frequencies. *p* value for Chi‐square test (with continuity correction when 2 × 2). Statistically significant associations are presented in bold (*p* < .05). High‐risk CVD: Systematic COronary Risk Evaluation (SCORE) ≥3; others: SCORE ≤2.

**TABLE 2:** Sociodemographic and clinical features of all participants and according to the cardiovascular disease (CVD) relative risk, for quantitative variables

Variables	All participants		Relative risk CVD				*p*
			High risk		Others		
	*M*/Mdn	±*SD*/IQR	*M*/Mdn	±*SD*/IQR	*M*/Mdn	±*SD*/IQR	
Age, years	31.00	15.00	38.50	15.00	30.00	13.00	**<.001**
Education, years of school	10.57	±2.49	9.69	±2.37	10.78	±2.45	.**003**
Seniority, years of contract	4.00	10	9.00	12.00	3.50	9.00	.**021**
SDur work days, hr:min	06:25	±01:27	06:01	±01:16	06:30	±01:28	.**021**
SDur free days, hr:min	08:22	±01:50	08:00	±01:33	08:28	±01:53	.074
Social jetlag, hr:min	01:45	01:57	02:00	02:03	01:40	02:04	.070
Chronotype, hr:min	03:54	03:54	4:18	03:04	3:48	4:05	.426
Total cholesterol, mg/dl	182.00	94.00	259.50	19.00	172	63.00	**<.001**
BMI, kg/m^2^	23.90	4.9	23.9	4.9	24.10	4.9	.691
Fasting, hr:min	02:00	02:00	02:00	02:00	02:00	02:00	.504
BP systolic, mmHg	119.00	17.00	120.50	27.00	118.00	16.00	.102
BP diastolic, mmHg	74.00	11.00	78.00	15.00	74.00	12.00	.**003**
Heart rate, bpm	79.00	17.00	80.00	17.00	78.00	17.00	.061
Productivity, items/month	19 947	9 926	17 869	11 174	20 467	9 943	.**004**
Absenteeism, absences/year	0	1	0	1	0	1	.323

Abbreviations: BMI, body mass index; BP, blood pressure; bpm, beats/min; CVD, cardiovascular disease; IQR, interquartile range; *M*, mean; Mdn, median; *SD*, standard deviation; SDur, sleep duration.

Data are presented as *M* ± *SD* for normally distributed continuous variables, and as Mdn and IQR for non‐normal distributions. Student *t* test *p* value for continuous normally distributed variables or Mann–Whitney *U* test for variables not normally distributed. Statistically significant associations are presented in bold (*p* < .05). Fasting represents the time elapsed (hr) since last meal when blood was collected for cholesterol analysis. Chronotype = mid‐sleep time on free days, sleep corrected (MSF_sc_). High‐risk CVD: Systematic COronary Risk Evaluation (SCORE) ≥3; others: SCORE ≤2.

### 2.2 Procedures and variables

Our observational study had a cross‐sectional design. Data collection took place between February and April 2019 in the medical office at the workplace, during the employees’ respective work hours. A self‐administered questionnaire was answered on a laptop, in the presence of a researcher, which collected subjective information about health, sleep, work, and sociodemographic features.

The Portuguese variant of the MCTQ (MCTQ^PT^) (Reis et al., 2020) was used to compute absolute SJL, individual chronotype (mid‐sleep time on free days, sleep corrected [MSF_sc_]) and sleep duration on both workdays and work‐free days. Chronotyping is based on the MSF, which is corrected for potential compensatory sleep resulting from sleep deprivation during the workweek, i.e. MSF_sc_. This correction is made by calculating the average sleep duration across the entire week (SDweek) and then correcting the MSF by subtracting half of the over‐sleep (for detail on computation see (Roenneberg et al., 2019). An MCTQ‐derived chronotype (MSF_sc_) is expressed in local time and can only be calculated if participants report not using alarm clocks on work‐free days, therefore our sample, considering this parameter was reduced to 229 participants (*n* = 121 morning, *n* = 88 evening, *n* = 20 night schedule). Although study participants are considered shift‐workers, as they work atypical schedules, these workers perform fixed (non‐rotational) shifts. Therefore, we use the regular MCTQ and not the MCTQ for Shift‐Workers (MCTQ‐shift) (Juda et al., 2013), as the adaptation created for chronotyping shift‐workers is primarily related to the changing nature of rotational shift‐work, not applicable here. SJL is a continuous variable measured in hours, quantified by calculating the absolute difference between the mid‐sleep point on work days (MSW) and MSF: SJL abs = |MSF‐MSW| (Roenneberg et al., 2019). Cut‐offs of 1 and 2 hr have been used in some epidemiological studies regarding SJL (Castilhos Beauvalet et al., 2017). In our study, SJL was analysed both as a continuous variable and as categorical, in an ordinal manner (≤2, 2–4 hr, ≥ 4 hr). Short sleep duration was defined as ≤6 hr for workdays (Luckhaupt et al., 2010). Sleep quality was assessed using the self‐rating question of the Portuguese version of the Pittsburgh Sleep Quality Index (PSQI‐PT) (Gomes et al., 2018). The answer was dichotomised into good versus poor sleep quality from the original 4‐item Likert scale (very good and good versus poor and very poor).

Sociodemographic characteristics included: sex, age, and education (years of school). Work information provided by the company included: seniority (number of years working at the company), absenteeism (total number of absences from work, in the previous calendar year) and productivity (mean value of picked items/month, in the previous calendar year). The CVD risk factors included: smoking status (currently smoker versus never smoker and former smoker), number of alcohol drinks consumed/day, number of caffeinated drinks consumed/day, and self‐reported weight and height (to calculate BMI). Recommended levels of physical exercise were defined as ≥150 min/week determined by two questions: the number of days doing leisure exercise/week and the duration of each session, according to National guidelines (Mendes et al., 2020). Recommended levels of drinks were set at ≤1 alcoholic drink/day and ≤3 caffeinated drinks/day (Thomas & Hodges, 2020; Williams et al., 2018). Objective health data was measured by a medical doctor and included TC, BP, and HR. The TC was assessed in non‐fasting capillary blood samples, as approved by recent guidelines (Mach et al., 2020), with Wellion LUNA Duo™ according to the manufacturer’s instructions. Although fasting was not required, the time elapsed since the last meal was recorded to assess the possibility of biased results in cholesterol levels. Hypercholesterolaemia was defined as TC ≥190 mg/dl (Piepoli et al., 2016). BP and HR were evaluated in a seated position, after 5 min of rest, using an automatic device (Easy Rapid PIC solutions™). Three measurements were taken, with ≥1 min rest in between, and the average of the last two was considered for the analysis, following the National survey protocol (INSA, 2016). Hypertension was defined as a systolic BP of ≥140 mmHg or diastolic BP of ≥90 mmHg (Williams et al., 2018).

**FIGURE 1:**
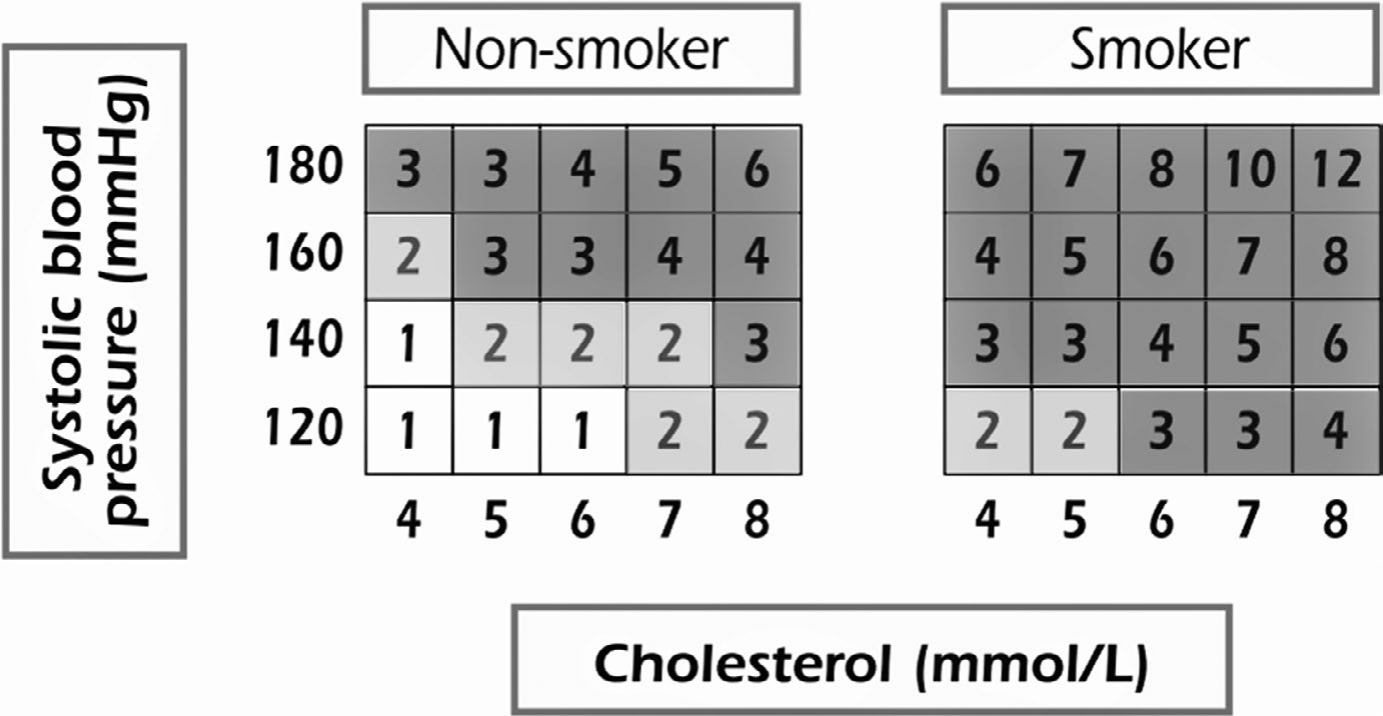
Relative risk chart derived from Systematic COronary Risk Evaluation (SCORE), adapted from 2016 European Guidelines on cardiovascular disease (CVD) prevention in clinical practice (Piepoli et al., 2016). SCORE = 1: low‐risk CVD (white); SCORE = 2: double the CVD risk (light grey); SCORE ≥3: at least triple the risk of CVD (high‐risk CVD; dark grey). Conversion of cholesterol units (mmol/L → mg/dl; 8 = 310; 7 = 279; 6 = 230; 5 = 190; 4 = 155)

Cardiovascular risk was defined using the relative risk chart derived from the SCORE (Figure 1) (Piepoli et al., 2016). This risk estimation is based on the interaction of main modifiable risk factors: smoking, SBP and TC values. A relative risk of 1 means an ideal combination of risk factors (i.e. low CVD risk), while a relative risk of 2 means double the risk, and so on. As there are no universal thresholds and the intensity of interventions should increase with increasing risk estimation, we define three relative risk categories (SCORE = 1, SCORE = 2, SCORE ≥3) (Pereira et al., 2014) and focussed on the high‐risk CVD group (SCORE ≥3) versus others (SCORE ≤2).

### 2.3 Statistical analyses

Descriptive statistics are provided for all participants and according to the CVD relative risk, i.e. high‐risk (SCORE ≥3) versus others (SCORE = 1 and SCORE = 2), as relative and absolute frequencies for categorical variables, as means and standard deviations (±*SD*) for normally distributed continuous variables, and as medians and IQRs for non‐normal distributions (Tables 1 and 2). Normality of data was tested using Shapiro–Wilk test.

For the bivariate analyses, differences across CVD risk categories (high‐risk versus others), were assessed using the Student *t* test (for continuous normally distributed variables) or Mann–Whitney *U* test (for non‐normally distributed variables), and the chi‐square test for categorical variables (Tables 1 and 2).

**FIGURE 2:**
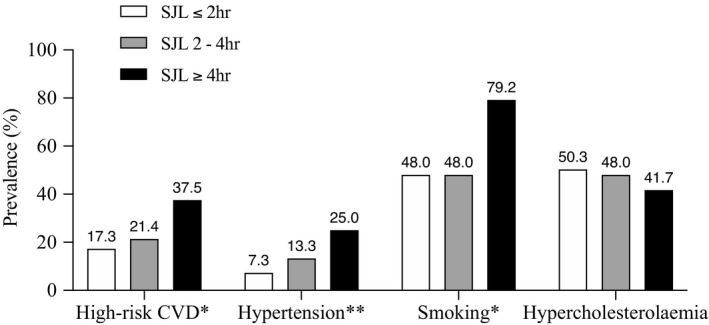
Prevalence of cardiovascular disease (CVD) outcomes per social jetlag (SJL) categories. Prevalence of high‐risk CVD (relative risk estimation; Systematic COronary Risk Evaluation [SCORE] ≥3), hypertension (blood pressure ≥140 or 90 mmHg), smoking (current smoker) and hypercholesterolaemia (total cholesterol ≥190 mg/dl) within ordinal SJL categories (≤2, 2–4, ≥4 hr). *p < .05 **p < .01. *p* value for linear‐by‐linear association chi‐square test; high‐risk CVD (*p* for trend = .035*), hypertension (*p* for trend = .006**), smoking (*p* for trend = .043*) and hypercholesterolaemia (*p* for trend = .439)

The chi‐square for trend (linear‐by‐linear association test) was used to assess the existence of a linear trend between ordinal categories of SJL (≤2, 2–4 hr, ≥4 hr) and poor sleep quality and cardiovascular outcomes (Figure 2). Also, among these SJL categories, the Kruskal–Wallis test was used to assess differences considering corporate data, namely productivity and absenteeism mean values.

Variables in the bivariate association with a *p* value < .20 (Aguiar, 2007) and/or relevant in the pathophysiological pathway were considered for further evaluation in multivariate statistical models. The relationship between SJL (as continuous variable) and being in the high‐risk CVD group was analysed through logistic binary regression, using generalised linear models. Smoking, BP and cholesterol were not included in the regression model as these parameters were already part of the SCORE estimation. To further explore the relationship between SJL and CVD risk, cumulative adjusted models were performed (Table 3). Model 1 represents the unadjusted association (crude odds ratio [OR]), while other models represent adjusted associations. In the second model (Model 2), we adjusted for main sociodemographic characteristics (sex, age [≤25, 25–40, ≥40 years] and education). In Model 3, we adjusted for additional CVD risk factors (excessive weight [BMI ≥25 kg/m^2^], insufficient exercise [<150 min/week], excess of alcohol [>1 drink/day], and caffeine [>3 drinks/day]). In Model 4, we adjusted for sleep parameters (sleep quality [poor] and sleep duration on workdays). Finally, in Model 5, we also adjusted for work characteristics (work schedule [morning, evening, night], seniority and productivity). Given the wide scale of the productivity variable, we transformed values by dividing per 1,000 (per each thousand), for the logistic regression analysis. The quality of the statistical models was assessed through the: (a) Hosmer and Lemeshow test (HL), with a *p* value > .05 identifying a good discriminatory ability; (b) area under the curve (AUC) for predicted probabilities, which a cut‐off of ≥0.7 being considered an acceptable prediction power and a value ≥0.80 being considered good (Aguiar, 2007); and (c) Akaike’s information criterion (AIC), where the lower the value, the lower the relative amount of information lost, therefore the better the model. Statistical analysis was performed using *Statistical Package for the Social Sciences* software (SPSS, version 26) and the level of significance considered for all statistical tests was 5%.

**TABLE 3:** Odds ratios (95% confidence intervals) for high risk of cardiovascular disease according to social jetlag, sociodemographic, lifestyle, sleep, and occupational features

Variables	Model 1		Model 2		Model 3		Model 4		Model 5	
	OR (95% CI)	*p*	OR (95% CI)	*p*	OR (95% CI)	*p*	OR (95% CI)	*p*	OR (95% CI)	*p*
Social jetlag hours	1.18 (1.01–1.39)	.**037**	1.23 (1.03–1.45)	.**019**	1.28 (1.06–1.54)	.**009**	1.25 (1.03–1.51)	.**022**	1.31 (1.02–1.68)	.**033**
Sex										
Female	‐	‐	Ref.		Ref.		Ref.		Ref.	
Male	‐	‐	0.68 (0.37–1.24)	.206	0.62 (0.31–1.25)	.185	0.58 (0.29–1.19)	.140	0.46 (0.20–1.06)	.070
Age, years										
≤25	‐	‐	Ref.		Ref.		Ref.		Ref.	
25–40	‐	‐	1.99 (0.80–4.98)	.139	1.97 (0.74–5.19)	.173	1.84 (0.69–4.94)	.227	2.42 (0.78–7.54)	.126
≥40	‐	‐	4.83 (1.90–12.30)	.**001**	4.93 (1.79–13.61)	.**002**	4.51 (1.61–12.61)	.**004**	6.10 (1.66–22.34)	.**006**
Education, years of school	‐	‐	0.87 (0.76–0.98)	.**027**	0.88 (0.77–1.01)	.071	0.88 (0.77–1.01)	.063	0.85 (0.73–0.99)	.**041**
BMI, kg/m^2^										
<25	‐	‐	‐	‐	Ref.		Ref.		Ref.	
≥25	‐	‐	‐	‐	0.63 (0.32–1.20)	.186	0.57 (0.28–1.16)	.121	0.47 (0.22–1.02)	.057
Exercise, min/week										
≥150	‐	‐	‐	‐	Ref.		Ref.		Ref.	
<150	‐	‐	‐	‐	6.50 (1.77–23.85)	.**005**	6.65 (1.83–24.14)	.**004**	4.61 (1.25–16.97)	.**022**
Alcohol, drink/day										
≤1	‐	‐	‐	‐	Ref.		Ref.		Ref.	
>1	‐	‐	‐	‐	0.87 (0.29–2.56)	.800	0.73 (0.24–2.23)	.581	1.05 (0.30–3.68)	.937
Caffeine, drinks/day										
≤3	‐	‐	‐	‐	Ref.		Ref.		Ref.	
>3	‐	‐	‐	‐	3.29 (1.65–6.56)	.**001**	3.46 (1.70–7.03)	.**001**	2.37 (1.09–5.12)	.**029**
SQua										
≥Good	‐	‐	‐	‐	‐	‐	Ref.		Ref.	
≤Poor	‐	‐	‐	‐	‐	‐	0.66 (0.31–1.39)	.275	0.64 (0.29–1.43)	.275
SDur workdays, hr	‐	‐	‐	‐	‐	‐	0.79 (0.61–1.03)	.081	0.82 (0.62–1.09)	.179
Schedule										
Morning	‐	‐	‐	‐	‐	‐	‐	‐	Ref.	
Evening	‐	‐	‐	‐	‐	‐	‐	‐	1.51 (0.60–3.78)	.380
Night	‐	‐	‐	‐	‐	‐	‐	‐	1.03 (0.26–4.05)	.963
Seniority, years of contract	‐	‐	‐	‐	‐	‐	‐	‐	0.98 (0.92–1.06)	.678
Productivity, per each 1,000	‐	‐	‐	‐	‐	‐	‐	‐	0.95 (0.91–0.99)	.**027**

Abbreviations: AIC, Akaike’s Information Criterion; AUC, area under the receiver operating characteristic curve; BMI, body mass index; CI, confidence interval; HL, Hosmer and Lemeshow test; OR, odds ratio; Ref., reference category; SDur, sleep duration; SQua, sleep quality.

Statistically significant associations are presented in bold (*p* < .05). **Model l** (unadjusted): Social Jetlag (*n* = 301; AIC = 180,125; AUC = 0.575; HL = 0.594); **Model 2**: Social jetlag adjusted for sex, age (≤25, 25–40, ≥40 years) and education (years of school) (*n* = 301; AIC = 277.845; AUC = 0.724; HL = 0.969); **Model 3**: Model 2 plus BMI (<25, ≥25 kg/m^2^), exercise (≥150, <150 min/week), alcohol (≤1, >1 drink/day) and caffeine (<3, >3 drinks/day) (*n* = 275; AIC = 249.933; AUC = 0.783; HL = 0.524); **Model 4**: Model 3 plus sleep quality (≥good, ≤bad), sleep duration on work days (*n* = 275; AIC = 250.454; AUC = 0.795; HL = 0.847); **Model 5** (final model): Model 4 plus work schedule (early, evening, night), seniority (years of contract) and productivity (per each 1,000 items/month) (*n* = 239; AIC = 227.695; AUC = 0.800; HL = 0.603). High risk of cardiovascular disease: Systematic COronary Risk Evaluation (SCORE) ≥3.

## 3 RESULTS

After excluding 49 workers (part‐time contracts, <3 months within the schedule, additional job, working standard schedules, extreme outliers and no blood collection; see methods) the final sample comprised 301 blue‐collar workers working permanent morning, evening, or night shifts. (Tables 1 and 2). Overall, 56% were males, the mean (*SD*, range) age was 33 (9.4, 18–57) years (73% aged <40 years) and 87.8% of the workers went to school for ≤12 years. The most frequent schedule was the morning (50.8%), followed by the evening (39.9%) and the night (9.3%), which was representative for the company’s workforce. The mean (*SD*) seniority of employees (time working at the company) was 6.1 (5.6) years (median 4 years; 30% ≤1 year). Regarding reported health behaviours: 79.6% of workers did not reach the recommended amount of physical exercise, 50.5% were smokers at the time of the study, 31.9% reported poor sleep, 29.6% drank more than three caffeinated drinks per day, and 9.6% drank more than one alcoholic drink per day. Reported mean (*SD*) sleep duration was 6:25 (1:27) hr on workdays (40% slept ≤6 hr) and 8:22 (1:50) hr on work‐free days. The mean (*SD*) SJL was 1:57 (1:38) hr (median 1:45 hr; 59.4% experienced SJL ≤2 hr and 8% SJL ≥4 hr). In terms of chronotype, mean (*SD*) chronotype (MSF_sc_) was 4:40 (2:46) (median 03:54). Concerning CVD risk, 39.5% (*n* = 119) of the participants fell into the low‐risk CVD category (SCORE = 1), 40.2% (*n* = 121) had twice the risk (SCORE = 2) and 20.3% (*n* = 61) had three‐times the risk or higher (SCORE ≥3: high‐risk CVD group).

In the bivariate analysis, workers in the high‐risk group (SCORE ≥3), compared to others (SCOREs 1 and 2), were significantly older (*p* < .001), had longer contracts (*p* = .021), were less educated (*p* = .003), slept on average shorter during workdays (*p* = .021), and had lower productivity (*p* = .004). This trend was also apparent when calculating prevalence, with the high‐risk group showing an increased frequency of short sleep on workdays (≤6 hr/day, *p* = .007), insufficient exercise (<150 min/week; *p* = .002) and caffeine abuse (≥3 drinks/day, *p* < .001). Notably, these workers in the high‐risk group (SCORE ≥3), had the same sleep duration on work‐free days (*p* = .074) compared to the other SCORE groups, and they also did not differ in the amount of SJL (*p* = .070) or on their chronotypes (*p* = .426) (Tables 1 and 2). As the SCORE already considers smoking, hypercholesteraemia and hypertension, these aspects were not surprisingly higher in the high‐risk group (all *p* ≤ .005). Differences found in cholesterol were unlikely due to differences in fasting, as the time elapsed from last meal was not significantly different among groups (*p* = .504).

Despite the bivariate results, we found significant linear trends for adverse cardiovascular outcomes when considering SJL as an ordinal qualitative variable (Figure 2). Considering the modifiable risk factors included in the SCORE, the prevalence of hypertension (*p* = .006) and smoking (*p* = .043) increased together with SJL. This was also the case for high CVD risk (*p* = .035). Only hypercholesterolaemia showed no significant trend (*p* = .439). For smoking, the prevalence increased exponentially among workers with SJL ≥4 hr (79.2%). Poor sleep quality and the three SJL categories (≤2 hr, 31.3%; 2–4 hr, 31.6%; ≥4 hr, 37.5%) were not associated (*p* = .638). Regarding corporate outcomes, such as productivity and absenteeism, no significant associations were found with SJL categories. Yet, despite lack of significance (*p* = .168), the highest mean productivity (18,995; for quantification, see methods) was found among workers with SJL ≤2 hr, followed by workers with 2–4 hr of SJL (17,297), and then workers with ≥4 hr of SJL (16,966). There was also no significant association between the number of absences from work and SJL categories (*p* = .165).

Most participants who experienced ≥4 hr of SJL were night workers (54.2%) followed by morning workers (41.7%) and just 4.2% who worked the evening shift. In agreement with this trend, most participants experiencing ≤2 hr of SJL worked the evening shift (58.0%), followed by 35.9% in the morning shift and just 6.1% working the night shift. Morning workers had the shortest sleep on workdays (mean [*SD*] 5:55 [1:12] hr), followed by night workers (mean [*SD*] 6:19 [1:24] hr) and evening (mean [*SD*] 07:06 [1:31] hr) (*p* < .001) (data not shown). In terms of chronotype, morning workers had the earliest average MSF_sc_ (mean [*SD*] 3:03 [1:16]), followed by evening workers (mean [*SD*] 6:17 [2:34]) and night workers (mean [*SD*] 7:29 [3:51]) (*p* < .001). We found a significant difference on chronotype for morning workers (*p* < .001), but no difference between evening and night workers (*p* = .535).

Logistic regression analyses showed that the odds of belonging to the high‐risk CVD group increased as the SJL increased, considering its continuous nature (OR 1.18, *p* = .037; Model 1: unadjusted; Table 3). To scrutinise this finding, cumulative models were performed to explore the possible links between SJL and CVD risk. We first assessed potentially confounding by sociodemographic features adjusting for age, sex, and education (Model 2). Model 2 showed a significant role for age (≥40 years; OR 4.83, *p* = .001), SJL (OR 1.23, *p* = .019), and education (OR 0.87, *p* = .027). Then, we consider the influence of other lifestyle risk factors that are not considered in the SCORE such as excessive weight, insufficient exercise, alcohol, and caffeine abuse (Model 3). Model 3 showed an increased risk again for SJL (OR 1.28, *p* = .009), older age, insufficient exercise (OR 6.50, *p* = .005) and caffeine abuse (OR 3.29, *p* = .001). Furthermore, we corrected the association for sleep variables, namely sleep quality and sleep duration on workdays (Model 4). Model 4 again showed an increased risk associated with SJL (OR 1.25, *p* = .022), older age, insufficient exercise, and caffeine abuse. Finally, we included specific work characteristics such as, schedule type, seniority, and productivity (Model 5).

For the final model (Model 5), the odds of having a high CVD risk increased >30% (OR 1.31, *p* = .033) with each additional hour of SJL, even after adjusting for all major confounding factors. In addition to SJL, the following factors were found to be independently associated with a greater chance of high CVD risk: age ≥40 years (OR 6.10, *p* = .006), insufficient exercise (OR 4.61, *p* = .022), and caffeine abuse (OR 2.37, *p* = .029). In contrast, education (OR 0.85, *p* = .041) and productivity (OR 0.95, *p* = .027) were associated with decreased odds of being in the high‐risk CVD group. Workers with higher productivity levels were associated with reduced chances of high CVD risk as there is evidence that for each added 1,000 [items/month] in productivity, the chances of high CVD risk reduces by ~5%. This final model was the one that better fits the data (lower AIC; AIC = 227.695) and had the best predictive power (≥80%; AUC = 0.800) and calibration (HL = 0.603). Overall, we found that SJL was an independent risk factor for cardiovascular health, as the positive association between SJL and high‐risk CVD group remained significant in all regression analyses, encompassing 31% higher risk in the most adjusted analysis.

## 4 DISCUSSION

The present study investigated the association between SJL and cardiovascular health, in a real‐life setting of blue‐collar workers following permanent atypical schedules. Our present results suggest that higher levels of SJL are associated with worse cardiovascular parameters. A higher degree of SJL was associated with greater odds of being in the high‐risk CVD group, after adjusting for sociodemographic, lifestyle, sleep, and work variables. Also, a significant trend was found for the prevalence of hypertension, smoking and high CVD risk, when considering SJL as an ordinal variable.

More than 40% of our population experienced >2 hr of SJL, a much higher proportion than reported to date (Koopman et al., 2017; Rutters et al., 2014). Only one study found a mean of 4:36 hr SJL among shift‐workers compared to 1:22 hr SJL among day workers (Yong et al., 2016). In our present population, only 8% experienced similarly high SJL (≥4 hr), which was associated with the highest prevalence of smoking (79.2%), hypertension (25.0%), and high CVD risk (37.5%). This is, to our knowledge, the first study identifying SJL as a risk factor for hypertension (Makarem et al., 2020). As for smoking, the increase of smoking prevalence along with SJL was already described (Wittmann et al., 2006). Night work is the most health‐challenging, and as its timing is furthest apart from that of usual social life and usual circadian entrainment, it is not surprising that ~55% of the night‐shift‐workers in our population experienced ≥4 hr of SJL. The remaining 45% of this group are presumably late chronotypes. In all, ~40% of the morning shift‐workers in our present population also experienced ≥4 hr of SJL, and in this case, the remaining 60% are presumably early chronotypes. Considering sleep duration on workdays, only evening workers achieved an average value (mean [*SD*] 07:06 [1:31] hr) in agreement with the 7–9 hr of sleep recommended for adults (Watson et al., 2015), while morning workers could not even achieve an average of 6 hr (mean [*SD*] 5:55 [1:12] hr). Our present findings in permanent shift‐workers mirrors those reported for rotational ones (Juda et al., 2013), where night and morning shifts are especially challenging for sleep and health.

The CVD prevalence was overall low in our population (e.g. hypertension ≈10%), which could be explained by its relatively young age (73% aged <40 years), as well as by the “healthy shift‐worker effect” (Moreno et al., 2019), which hypothesises that relative stable health in shift‐workers is due to a selection towards those who tolerate it. The relative risk SCORE focusses on modifiable risk factors, a rationale that can be applied to everyone, even young people who show no substantial absolute CVD risk. Recent guidelines recommend that CVD prevention should apply a lifetime approach because, most CVD‐related deaths are associated with absolute lower risk, simply because they are more numerous compared with high‐risk individuals (Mach et al., 2020). While an increasing number of workers have to follow non‐standard schedules, there is also growing scientific evidence that associates shift‐work to deleterious health outcomes (Rivera et al., 2020). Recommendations regarding the circadian physiology are already defined to minimise the impact of atypical schedules on health, e.g. avoid morning shift or heavy workload in the circadian nadir (04:00–07:00 hours), shift duration <12 hr or clockwise rotation among rotating shifts (Czeisler et al., 1982; Drake et al., 2011); however, worker’s chronotype is still not systematically considered on schedule designs. Evidence on the role of chronotype on sleep and wellbeing across real‐life rotational workers have already been shown by an observation study by Juda et al. (2013), but also in an intervention study, with a chronotype‐adjusted shift schedules, conducted by Vetter et al. (2015). Our present study supports the evidence that SJL and individual chronotype should be considered in assigning shifts to workers. It also contributes to our understanding of the link between health and circadian misalignment, specifically concerning cardiovascular, physiological, and behavioural risk factors.

Our multivariate analysis showed that, in addition to SJL, older age, insufficient exercise, and caffeine abuse were independently associated with higher odds of having a high CVD risk. Age and insufficient exercise are well established CVD risk factors (Piepoli et al., 2016), as is caffeine abuse (via blood pressure) and/or as a confounder of smoking (Thomas & Hodges, 2020). In contrast, education and productivity were associated with decreased risk. In a representative sample of the Portuguese population, lower education was also associated with an increased prevalence of high and very‐high CVD risk (Gaio et al., 2020). In our present study, higher productivity was associated with lower risk, possibly via the protective effect of higher physical activity. Nevertheless, others showed that men (not women), with high‐physical‐activity jobs had decreased health (Coenen et al., 2018), but again that could be confounded by socioeconomic status and education. Bidirectional associations are also possible. Thus, the link between higher productivity and reduced CVD risk still needs further research.

One strength of the present study is the homogeneity of the sample, which allows comparisons between subjects with reduced confounders. Another strength, is that we actually collect BP and cholesterol data with a validated and standardised procedure, and not used information from medical records. Finally, we had access to productivity and absenteeism data.

The present study also had some limitations: our cross‐sectional design excludes causal inferences (future experimental studies will have to elucidate the causal pathways allowing an evidence‐based reduction of SJL and thereby minimisation of CVD risk); sleep and anthropometric indicators were self‐reported (future studies should use actiometry and assess objectively anthropometric measurements) (Ancoli‐Israel et al., 2003); finally, BP was measured in one occasion (future studies should use 24‐hr ambulatory monitoring allowing valuable over‐night measurements (Williams et al., 2018)).

The present study is the first to indicate that reducing SJL potentially reduces CVD risk. Our findings encourage future cardiovascular‐health studies to include sleep and circadian qualities as they will be critical to understand and prevent negative health impacts of shift‐work.

## CONFLICT OF INTEREST

TR is the founder and Chief Scientific Officer of the company Chronsulting UG and consults several other companies (Vanda Pharmaceuticals, Chiesi GmbH, jetlite GmbH, Condor Instruments, Salzgitter AG, PricewaterhouseCoopers, Weightwatchers, KGK Science Inc.); none of these activities created conflicts with the content of this paper. SGM, CR, TP, PN, CM have no conflict of interest to declare.

## AUTHOR CONTRIBUTIONS

SGM conceived the original idea, carried out the data collection and analyses, and took the lead in writing the manuscript. TP, CM, and TR contributed to the design and implementation of the study. CR, TP, PN, and TR gave substantial contributions to the analysis and interpretation of data and revising the manuscript critically for important intellectual content. TR developed the conceptual framework and the operationalization of the Munich Chronotype Questionnaire. All authors discussed the results and contributed to the final manuscript.

## Data Availability

The data that support the findings of this study are available on request from the corresponding author. The data are not publicly available due to privacy or ethical restrictions.
